# Trends in Outpatient Telemedicine Utilization Among Rural Medicare Beneficiaries, 2010 to 2019

**DOI:** 10.1001/jamahealthforum.2021.3282

**Published:** 2021-10-15

**Authors:** Michael L. Barnett, Haiden A. Huskamp, Alisa B. Busch, Lori Uscher-Pines, Krisda H. Chaiyachati, Ateev Mehrotra

**Affiliations:** 1 Department of Health Policy and Management, Harvard T. H. Chan School of Public Health, Boston, Massachusetts; 2 Department of Medicine, Brigham and Women’s Hospital, Boston, Massachusetts; 3 Department of Health Care Policy, Harvard Medical School, Boston, Massachusetts; 4 McLean Hospital, Belmont, Massachusetts; 5 RAND Corporation, Arlington, Virginia; 6 Department of Medicine, The Perelman School of Medicine at the University of Pennsylvania, Philadelphia; 7 Department of Medicine, Beth Israel Deaconess Medical Center, Boston, Massachusetts

## Abstract

**Importance:**

Little is known about how telemedicine use was evolving before the broad changes that occurred during the COVID-19 pandemic in 2020. Understanding prepandemic patterns of telemedicine use can inform ongoing debates on the future of telemedicine policy.

**Objective:**

To describe trends in telemedicine utilization among Medicare fee-for-service beneficiaries before the COVID-19 pandemic and the specialties of clinicians providing telemedicine.

**Design, Setting, and Participants:**

This was a cross-sectional study and descriptive analysis of telemedicine utilization by 10.4 million fee-for-service Medicare beneficiaries from 2010 to 2019. Data analysis was performed from June 6, 2019, to July 30, 2020.

**Main Outcomes and Measures:**

Rates of telemedicine utilization, characteristics of beneficiaries who received telemedicine in 2010 to 2019, and specialties of clinicians delivering telemedicine.

**Results:**

Of 10.4 million rural Medicare beneficiaries, telemedicine was used by 91 483 individuals (age ≥65 years, 47 135 [51.5%]; women, 51 476 [56.3%]; and White, 76 467 [83.6%] individuals) in 2019. In 2010 to 2019, telemedicine visits grew by 23.1% annually. A total of 0.9% of all fee-for-service rural beneficiaries had a telemedicine visit in 2019 compared with 0.2% in 2010. In 2019, there were 257 979 telemedicine visits or 34.8 visits per 1000 rural beneficiaries and most (75.9%) of these visits were for mental health conditions. Patients with bipolar disorder or schizophrenia (3.0% of rural beneficiaries) received 40% of all telemedicine visits in 2019. Some traditionally disadvantaged and underserved groups comprised a larger share of telemedicine users than nonusers in 2019, such as those dually insured with Medicaid (56.9% of users vs 18.6% of nonusers; adjusted odd ratio, 3.83; 95% CI, 3.77-3.89). In 2010 to 2019, telemedicine for mental health conditions shifted away from psychiatrists (71.2% to 35.8% of all telemedicine visits) to nonphysician clinicians, eg, nurse practitioners, psychologists, and social workers (21.4% to 57.2%). There was wide variation in telemedicine utilization in 2019 across counties: median (IQR), 16.0 (2.5-51.4) telemedicine users per 1000 beneficiaries). In 891 counties (29% of all US counties), at least 10% of beneficiaries with bipolar disorder or schizophrenia used a telemedicine service in 2019.

**Conclusions and Relevance:**

In this cross-sectional study of telemedicine utilization before the COVID-19 pandemic, there was sustained growth in telemedicine visits among rural beneficiaries covered by the Medicare program, especially care delivered by nurse practitioners and other nonphysician clinicians. The prepandemic model of telemedicine provided in local health care settings may be a viable modality to maintain in rural communities.

## Key Points

**Question:**

How was telemedicine used by rural Medicare beneficiaries in the decade prior to the COVID-19 pandemic?

**Findings:**

This cross-sectional study of 10.4 million rural Medicare beneficiaries found sustained annual compound growth in telemedicine use among rural beneficiaries covered by Medicare in 2010 to 2019, especially for care provided by nurse practitioners and other nonphysician clinicians. Medicare beneficiaries with serious mental illness (eg, bipolar disorder) used a disproportionate share of all telemedicine visits, with more than 1 in 10 beneficiaries using telemedicine annually in 891 counties in the US.

**Meaning:**

The findings of this cross-sectional study indicate that telemedicine use grew rapidly before the COVID-19 pandemic among Medicare beneficiaries with mental health conditions; the model of telemedicine provided in the local health care setting before 2020 may be a viable modality for continued use in rural communities.

## Teaser

This cross-sectional study describes telemedicine utilization trends among rural Medicare beneficiaries from 2010 to 2019 to ascertain if the model being used before the COVID-19 pandemic should continue to be used.

## Introduction

Telemedicine, the provision of clinical care via remote audiovisual telecommunications may increase access to care^1,2^ and improve health care quality,^3^ particularly for people living in rural and underresourced settings.^4,5,6^ During the COVID-19 pandemic, telemedicine use for office-based visits has surged.^7,8,9^ This growth has been facilitated by temporary regulatory and payment changes that have expanded coverage of telemedicine in the Medicare program.^10^ There is ongoing debate at both the state and federal levels on whether some or all of these changes should be made permanent.^11^

Before the pandemic there were many restrictions on the use of telemedicine within the Medicare program, such as requiring beneficiaries to receive telemedicine care via live (real time) videoconference (ie, no audio-only visits) and at a clinic or hospital in a rural area (ie, no home-based visits).^12^ These facilities providing the technology and space for telemedicine are known as an “originating site.” The SUPPORT Act, signed into law in 2018, relaxed some of these restrictions for the purposes of addiction treatment,^13^ while the 2021 Consolidated Appropriations Act expanded the range of beneficiaries eligible for telemedicine for mental health^14^; however, some barriers such as the requirement for in-person visits were retained in the latter legislation. Prior research describing utilization trends through 2017^15,16,17^ found that outpatient telemedicine use within the Medicare program was low and predominantly used for mental health diagnoses, with the highest rates of use among beneficiaries with serious mental illness, such as bipolar or psychotic disorders.

To our knowledge, there have been only a few descriptions of telemedicine use in the Medicare program during the years leading up to the COVID-19 pandemic.^18^ Understanding prepandemic patterns of telemedicine use can inform what the future may be like if previous restrictions return, and can help us to understand prepandemic disparities driven by the digital divide, that is, the gaps in availability of technology necessary for conducting telemedicine visits or the skills needed to use the technology.^19,20,21,22^ Telemedicine use before 2020 can also aid understanding of potentially successful models of telemedicine that were in regular use despite the tight restrictions.

To address the evidence gap in telemedicine use before 2020, this study examines and describes which conditions were being treated, the geographic variations, the patients most likely to use telemedicine, and which health care professionals were engaged in outpatient telemedicine in 2010 to 2019.

## Methods

The study was reviewed and approved by the institutional review board of Harvard Medical School. Informed consent was waived because only deidentified data were provided for the study analyses according to the Regulations for the Protection of Human Subjects (45 CFR §46). The study followed the Strengthening the Reporting of Observational Studies in Epidemiology (STROBE) reporting guideline.

### Data Sources and Study Sample

The study’s primary data source was Medicare Part B administrative claims for 2010 to 2019 for a 100% sample of fee-for-service Medicare beneficiaries residing in rural areas. The sample was limited to rural residents because before the pandemic, Medicare reimbursements were only for telemedicine visits hosted by a clinic or hospital.^23,24^ Similar to Medicare’s definition of rural eligibility,^23^ we defined rural beneficiaries as those residing in zip codes outside of a core-based statistical area or within an area assigned a rural-urban commuting area code of 4 to 10 (micropolitan areas to rural areas).^25^

### Identifying and Classifying Outpatient Telemedicine Visits

This analysis focused on telemedicine visits that could serve as alternatives or substitutes for in-person outpatient office visits. Outpatient telemedicine visits were defined as an evaluation and management visit with a place of service code of 02 (telemedicine) or with 1 of the following modifiers: GT (via interactive audio and video telecommunication systems); 95 (via synchronous, real-time, interactive audio and video telecommunication system); or GQ (via asynchronous telecommunications system). We did not include codes 99441 to 99443 (telephone evaluation and management visits) or codes for virtual check-ins or e-visits because they were not widely reimbursed as telemedicine visits before the COVID-19 pandemic.

We divided telemedicine visits into 2 mutually exclusive categories: mental health (including substance use disorders) and nonmental health. Mental health visits were defined as visits for a primary diagnosis of any mental health or substance use disorder (*International Classification of Diseases, Ninth Revision [ICD-9]*, codes 291.x, 292.x, 295.x-316.x, and *International Statistical Classification of Diseases and Related Health Problems, Tenth Revision [ICD-10]*, codes F10.x-F69.x, F80.x-F99.x), excluding disorders related to dementia, brain injury, and tobacco dependence. All other visits were defined as nonmental health, including the visits related to dementia, brain injury, and tobacco dependence that had been excluded from the mental health category.

### Beneficiary Characteristics and Cohorts

From annual Medicare enrollment records, we captured beneficiary characteristics, including age, sex, race or ethnicity, reason for Medicare eligibility, and any dual Medicare and Medicaid enrollment status. Data on race came from an imputed variable created by the Research Triangle Institute for Medicare.^26^ Using the beneficiaries’ zip code merged with US census data, we categorized beneficiaries as above or below 200% of the federal poverty limit using the median family income of their zip code of residence.

Given the predominance of telemedicine visits in any calendar year were for treatment of mental illness, we also classified beneficiaries into 3 mutually exclusive cohorts: severe mental illness (SMI; SMI cohort), mental health diagnosis but not SMI (mental health cohort), and no mental health diagnosis (nonmental health cohort). To meet criteria for a cohort in a given year, a beneficiary was required to have claims for at least 2 outpatient visits with an appropriate diagnosis code in any diagnostic field or 1 inpatient admission for a serious mental health condition or mental health condition using *ICD-9* (before September 2015) or *ICD-10* (after September 2015) system codes. The typical definition of SMI was based on degree of functional impairment.^27^ Given functional impairment was not available in administrative claims data, we opted for a conservative definition using typically disabling conditions, that is, schizophrenia and psychotic disorders (*ICD-9* codes 295 or 297 and *ICD-10* codes F20-F29) and bipolar I disorder (*ICD-9* codes 296.0, 296.1, 296.4-296.6, 296.7, 296.80, 296.81, 296.89, 296.9, 301.11, or 301.13 and *ICD-10* codes F30-F31 or F34.0). Other mental health conditions were defined as *ICD-9* codes 291, 292, and 295-316, excluding 310 (disorders related to brain damage), 305.1 (tobacco dependence), and 305.8 (antidepressant abuse); and *ICD-10* codes F10-F69, F80-F89, F90-F98, and F99, excluding F17 (tobacco dependence).

### Classifying Clinician Specialties

We classified the specialty of clinicians using the primary specialty associated with the National Provider Identifier Code. We examined telemedicine claims by physician and nonphysician clinicians. We defined primary care physicians (PCPs) as specializing in internal medicine, family medicine, geriatrics, or general practice. Because numerous specialties had small volumes of telemedicine visits, we grouped the top 8 specialties with the highest telemedicine volumes (excluding psychiatrists) into a specialist MDs category. We also grouped together nonphysician clinicians, including nurse practitioners (NPs), physician assistants (PAs), clinical social workers (SWs), and psychologists. Because of their distinct clinical profiles, we differentiated NPs into 2 groups: psychiatric mental health NPs (PMHNPs, the preferred title for the clinicians)^28^ and other NPs. The PMHNPs were defined as NPs who had used a mental health primary diagnosis code for more than 80% of their professional claims, whether these visits were in-person or telemedicine.

### Outcomes

The study’s main outcome was the quarterly count of telemedicine visits billed in Medicare claims during the time period of the study (2010-2019), overall and divided into the mutually exclusive cohorts: SMI, mental health, and nonmental health. We also examined the rate of telemedicine visits per 1000 Medicare beneficiaries to adjust for the changing number of Medicare beneficiaries. We examined quarterly counts and per-capita visit rates nationally and by several patient-level sociodemographic subgroups and clinician types. We examined the distribution of telemedicine visits across separate patient cohorts by comparing the proportion of all telemedicine visits used by the 3 mutually exclusive patient cohorts (SMI, mental health, and nonmental health) with their distribution in the overall Medicare beneficiary population.

### Statistical Analyses

We described telemedicine visits among rural Medicare beneficiaries in 2010 to 2019 and variation in telemedicine use by beneficiary characteristics in 2010 and 2019 among beneficiaries who did and did not have any telemedicine encounters in those years. We used multivariable logistic regression models to estimate the beneficiary factors independently associated with telemedicine use in 2019, the time period immediately before the COVID-19 pandemic. The unit of analysis was the beneficiary. The outcome was whether the beneficiary had a telemedicine visit in 2019; the variables in the model were age, sex, race or ethnicity, rural-urban commuting area code for the beneficiary’s zip code, and reason for Medicare enrollment (disability, end-stage renal disease, or age). Data analyses were performed June 6, 2019, to July 30, 2020, using SAS, version 9.4 (SAS Institute Inc). Statistical tests were 2-tailed, and significance was defined as *P* ≤ .05.

## Results

###  Characteristics of Telemedicine Users

In 2019, of the 10.4 million rural Medicare beneficiaries, 91 483 individuals (age ≥65 years, 47 135 [51.5%]; women, 51 476 [56.3%]) used telemedicine; 6495 (7.1%) were Black, 76 467 (83.6%) were White, and 8555 (9.4%) were individuals of other race or ethnicity. The category “other race and ethnicity” came from an imputed variable created by the Research Triangle Institute for Medicare^26^ and included Asian, American Indian/Alaska Native/Pacific Islander, and Hispanic beneficiaries (Table).

**Table.** Characteristics of All Rural Medicare Beneficiaries With and Without a Telemedicine Visit in 2019

Characteristic	Telemedicine use		Telemedicine use, aOR (95% CI)
	No, %	Yes, %	
Total, No.	10 329 920	91 483	NA
Age, y			
<65	15.5	48.5	1.80 (1.76-1.84)
65-74	49.1	28.5	1 [Reference]
75-84	24.8	16.0	1.13 (1.11-1.16)
≥85	0.6	7.0	1.06 (1.03-1.09)
Race and ethnicity^a^			
Black	5.8	7.1	0.86 (0.83-0.88)
White	86.6	83.6	1 [Reference]
Other	7.6	9.4	0.95 (0.93-0.98)
Sex			
Men	48.2	43.7	1 [Reference]
Women	51.8	56.3	1.23 (1.21-1.24)
US census division			
New England	4.1	2.8	1 [Reference]
Middle Atlantic	6.4	2.6	0.62 (0.59-0.66)
East North Central	15.4	17.4	1.81 (1.74-1.89)
West North Central	13.1	23.6	3.32 (3.19-3.47)
South Atlantic	18.5	14.0	1.29 (1.23-1.34)
East South Central	12.0	10.1	1.17 (1.12-1.23)
West South Central	12.9	11.9	1.53 (1.47-1.6)
Mountain	8.2	8.9	2.08 (1.98-2.18)
Pacific	9.4	8.8	1.58 (1.51-1.66)
Medicare eligibility			
Age ≥65 y	74.7	38.0	1 [Reference]
Disability	24.8	61.4	2.09 (2.05-2.14)
End-stage renal disease	0.6	0.7	0.91 (0.84-0.99)
Median income, FPL^b^			
<200%	70.9	76.0	1 [Reference]
≥200%	29.1	24.0	1.09 (1.07-1.1)
Medicaid eligibility			
Yes	18.6	56.9	3.83 (3.77-3.89)
No	81.4	43.1	1 [Reference]

Abbreviations: aOR, adjusted odd ratio; FPL, federal poverty level.

^a^ Data on race and ethnicity came from an imputed variable created by the Research Triangle Institute for Medicare^26^ and “other” race and ethnicity included Asian, American Indian/Alaska Native/Pacific Islander, and Hispanic patients. Percentages may not total 100 owing to rounding.

^b^ Income was based on the median income within the zip code of the beneficiary’s residence per US census data. Zip codes representing 2.1% of beneficiaries could not be matched to census data.

In 2019, rural Medicare beneficiaries with a telemedicine visit were more likely than beneficiaries without a telemedicine visit to be covered by Medicare for a disability (61.4% vs 24.8%; adjusted odds ratio [aOR], 2.09; 95% CI, 2.05-2.14) and be dually insured by Medicaid (56.9% vs 18.6%; aOR, 3.83; 95% CI, 3.77-3.89). See eTable 1 in the Supplement for more details. Other traditionally disadvantaged groups comprised a larger share of telemedicine users than nonusers in 2019, such as Black beneficiaries (7.1% vs 5.8%); however, after adjusting for other demographic factors, this association switched direction (aOR, 0.86; 95% CI, 0.83-0.88).

### Outpatient Telemedicine Volume

**Figure 1.**
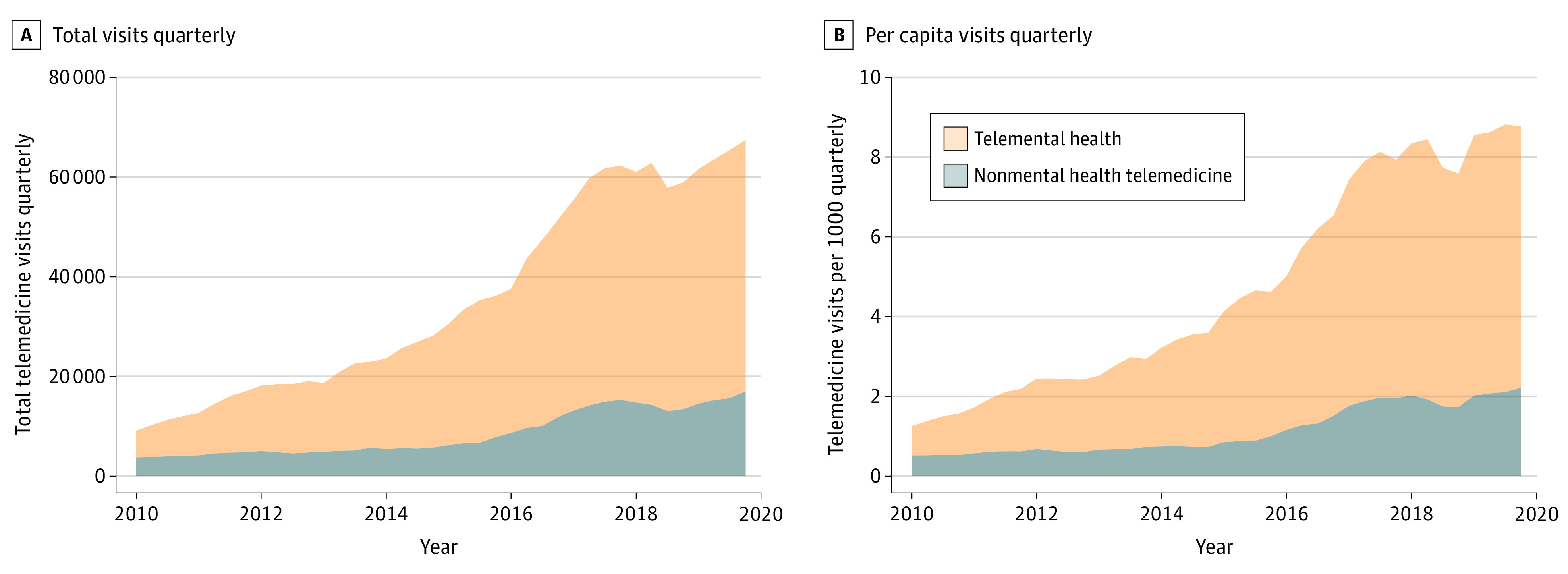
Trends in Quarterly Telemedicine Visits for Rural Medicare Beneficiaries, 2010 to 2019 Unadjusted counts of telemedicine visits quarterly (A) or telemedicine visits per 1000 rural Medicare beneficiaries quarterly (B) in 2010 to 2019. Mental health telemedicine visits (orange) and nonmental health telemedicine visits (gray) sum to the total of all telemedicine visits.

From 2010 to 2019, the number of telemedicine visits increased by an average of 23.1% per year (Figure 1). In 2010, there were 42 608 telemedicine visits, or 5.7 per 1000 rural beneficiaries annually (1.4 per 1000 quarterly); by 2019, the number had increased to 257 979 visits or 34.8 visits per 1000 rural beneficiaries annually (8.7 per 1000 quarterly). A total of 0.9% of all fee-for-service rural beneficiaries had a telemedicine visit in 2019 compared with 0.2% in 2010 (eTable 1 in the Supplement).

Growth in per-capita telemedicine use varied substantially by the users’ characteristics, with faster growth among beneficiaries with a disability, those who were Medicaid eligible, and those residing in higher-income zip codes (eFigure in the Supplement).

### Telemedicine Use by Condition and Cohort

**Figure 2.**
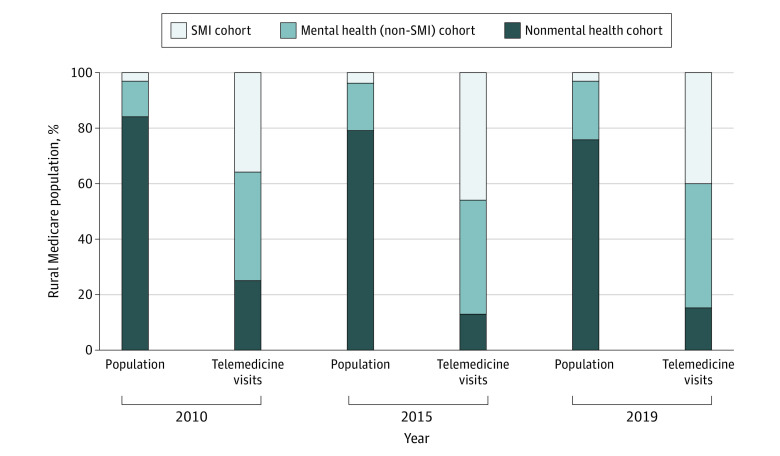
Distribution of Rural Medicare Population Subgroups Compared With Their Telemedicine Visit Use in 2010, 2015, and 2019 Proportion of rural Medicare beneficiaries (left bar) and proportion of all telemedicine visits used per year (right bar) by each of the 3 mutually exclusive groups: (1) patients with an SMI, (2) patients with a mental health diagnosis but not an SMI, and (3) all others with no defined mental health condition. Abbreviation: SMI, serious mental health illness (defined by degree of functional impairment^27^).

Mental health treatment represented most of the telemedicine use, totaling 195 705 visits (75.9%) of all telemedicine visits among the study sample in 2019. A disproportionate number of telemedicine visits were delivered to beneficiaries with SMI. In 2019, the SMI cohort represented only 3% of the rural population but used 40% of the telemedicine visits that year (Figure 2). In contrast, 76% of the rural Medicare population that was in the nonmental health cohort used only 15% of all the telemedicine visits. These patterns were stable during the 10-year period from 2010 to 2019.

### Telemedicine Volume by Clinician Specialty

**Figure 3.**
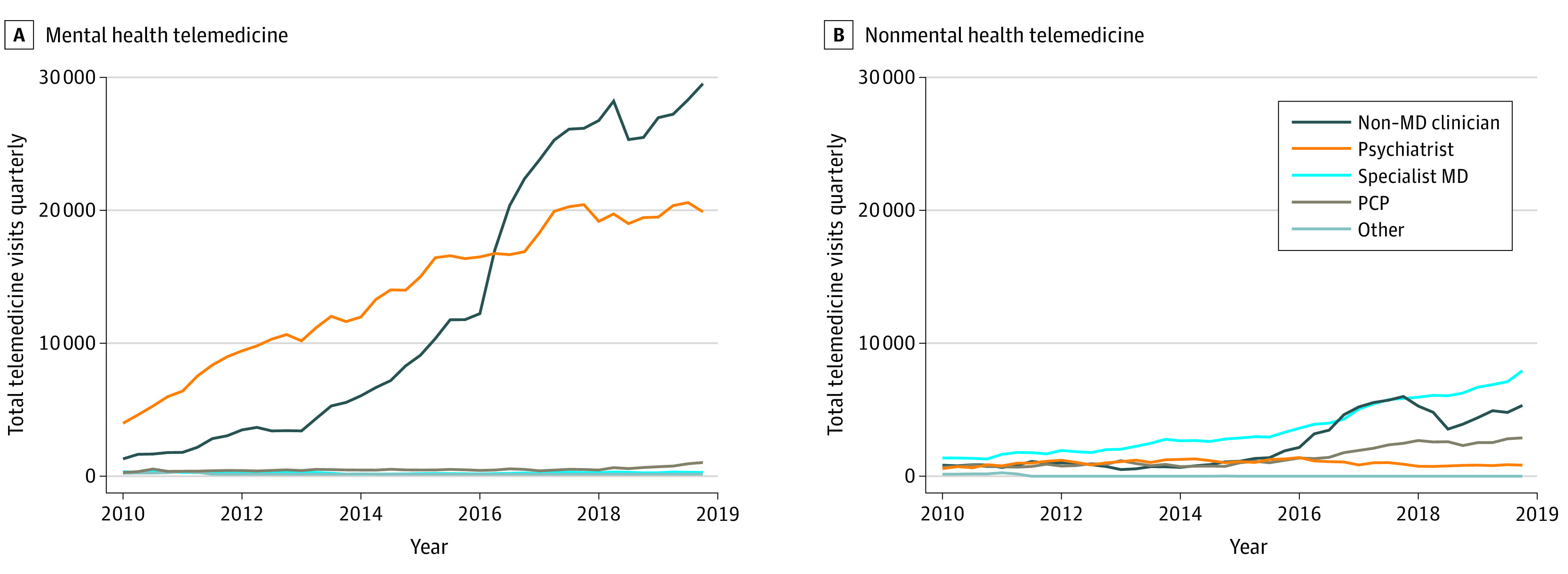
Trends in Telemedicine Visits for Rural Medicare Beneficiaries, by Clinician Specialty, 2010 to 2019 Trends in quarterly mental health (A) and nonmental health (B) telemedicine visits by clinician specialty. The Non-MD clinician category comprises care provided by nurse practitioners (NPs), mental health NPs, physician assistants, psychologists, and social workers; Specialist MD, all medical specialties other than primary care and psychiatry (most commonly sleep medicine, nephrology, and hematology-oncology); PCP, physicians specialized in internal medicine, family medicine, geriatrics, or general practice; and Other, all other practitioners (eg, dentists or unknown specialty).

As the volume of telemedicine grew from 2010 to 2019, the composition of clinicians providing these visits changed (Figure 3). In 2010, psychiatrists were providing most of the mental health telemedicine, delivering 19 343 visits or 71.2% of all the mental health telemedicine visits that year (eTable 2 in the Supplement). By 2019, psychiatrists delivered only 35.8% of all mental health telemedicine, while nonphysician clinicians provided 57.2% (up from 21.4% in 2010) telemedicine mental health care. Mental health NPs represented the largest volume of telemedicine clinicians among nonphysician clinicians, delivering 52 855 visits or 27.0% of telemedicine mental health visits in 2019. Psychologists and social workers also provided substantially more telemedicine in 2019 than they had in 2010 (3.4% and 0.8% in 2010 vs 14.9% and 8.6% in 2019, respectively).

Nonphysician clinicians also delivered more nonmental health telemedicine visits; from 2010 to 2019 their share of all telemedicine visits grew from 20.8% to 30.3% (Figure 3; eTable 2 in the Supplement). Although PCPs provided 17.3% of nonmental health telemedicine in 2019, the largest of any physician category, no other single specialty was dominant. Sleep medicine specialists were the largest specialty besides PCPs, delivering 3393 visits or 5.5% of all nonmental health telemedicine visits in 2019.

### Geographic Variation and Telemedicine Use

**Figure 4.**
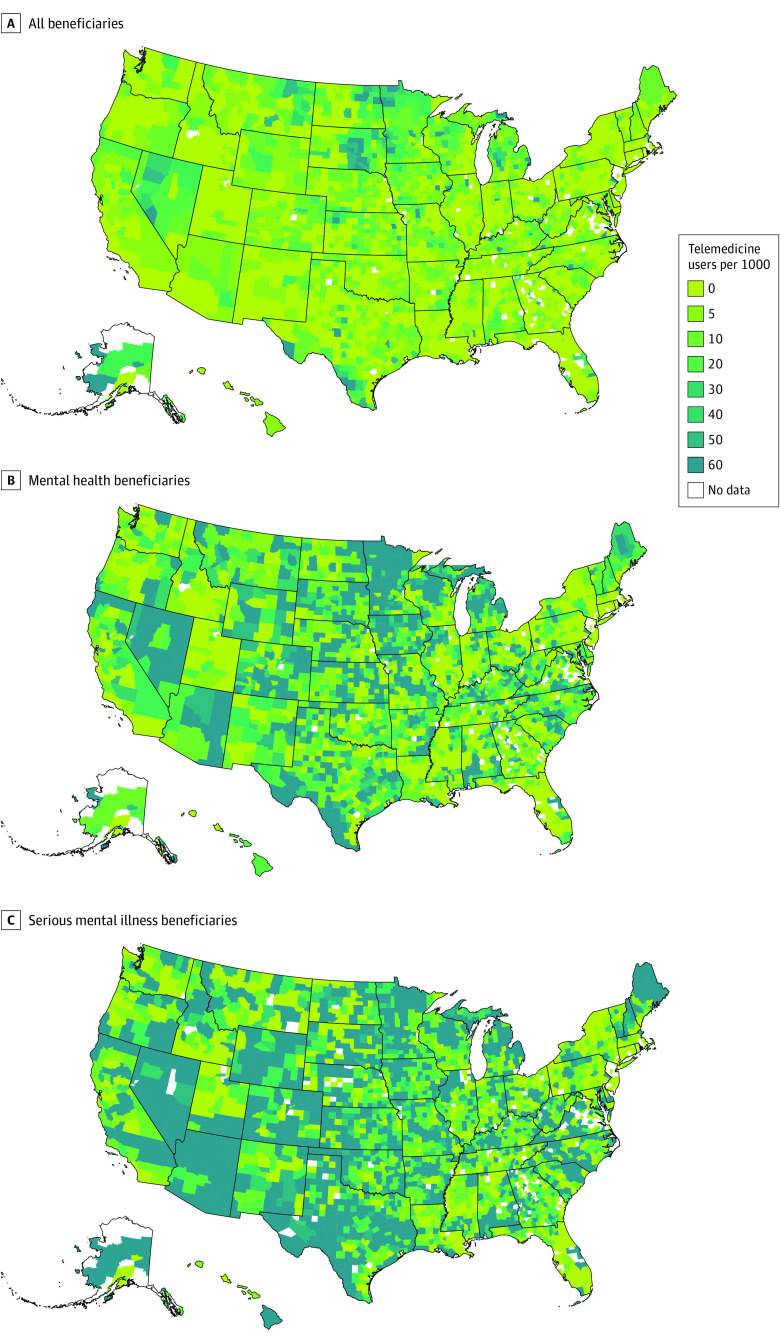
Telemedicine Use (All Visits), by Rural Medicare Beneficiaries and by County, 2019 County-level number of telemedicine users per 1000 Medicare beneficiaries in 2019. A, Telemedicine use rates for all Medicare beneficiaries in each county. B, Use per 1000 beneficiaries with a mental illness diagnosis but not an SMI. C, Use per 1000 beneficiaries with an SMI. Data were not available for counties with 11 or fewer eligible fee-for-service Medicare beneficiaries in both cohorts because of suppression of small cell sizes. Abbreviation: SMI, serious mental health illness (defined by degree of functional impairment^27^).

There was broad variation in the use of telemedicine by rural Medicare beneficiaries in 2019, with a county-level median (IQR) of 4.9 (0.7-12.4) beneficiaries with telemedicine use per 1000 beneficiaries (Figure 4). Among the mental health cohort, the median (IQR) county had 16.0 (2.5-51.4) telemedicine users per 1000 beneficiaries, and 28.6 (6.9-133.2) for the SMI cohort in 2019. In 296 (9.7%) of 3051 counties with more than 12 Medicare beneficiaries, at least 10% of the 105 890 beneficiaries in the mental health cohort used a telemedicine service in 2019, and in 891 (29.2%) of those counties, 10% or more of the 86 681 beneficiaries in the SMI cohort used telemedicine that year.

## Discussion

Before the COVID-19 pandemic, telemedicine had become an important component of health care delivery among the rural Medicare population, especially for mental health. Telemedicine use was concentrated among vulnerable populations including those with SMI, those dually eligible for Medicaid, and those residing in lower-income zip codes. These study findings suggest that prior to the COVID-19 pandemic, telemedicine was reaching, at least in part, across the digital divide for rural Medicare beneficiaries. Although this study describes patterns of care prior to the pandemic, the results on telemedicine shed light on important considerations for the ongoing debate regarding the future of telemedicine policy; specifically, that recent data show that during the pandemic, rural residents have been using telemedicine at much lower rates than urban residents.^7,29^

A decade of continuous and steady growth of telemedicine use in rural areas shows that the originating site facility-based telemedicine model was successfully engaging many disadvantaged rural patients. In several hundred counties, more than 1 in 10 rural Medicare beneficiaries with mental illness used telemedicine in 2019. These findings imply that the telemedicine originating site facilities, where beneficiaries travel to a clinical facility to participate in audiovisual telemedicine, were active centralized facilities for remote specialty access. Given the substantial concerns about disparities in broadband access and the gaps in technology literacy among older people in rural areas,^30,31^ this centralized model may have allowed patients without the necessary skills or technology at home to access specialty care. While the substantial policy focus on increasing access to broadband and technology in rural areas is long overdue, we also believe that we should create greater incentives to maintain or expand the originating site model within rural health facilities. Otherwise, underserved rural patients may lose an important lifeline for receiving remote health care.

Another key finding, consistent with prior evidence,^16,17,32^ was that telemedicine use was dominated by mental health care. This is not surprising given the suitability of mental health for telemedicine itself. Mental health care relies less on the physical examination than do other clinical disciplines, making virtual care a reasonable substitute for in-person care. Also, mental health professionals are in especially short supply in rural areas, so telemedicine can substantially broaden the range of clinicians who can deliver care in those settings. These findings suggest that future policy should prioritize maintaining telemedicine reimbursement and coverage for mental health care delivery to build on demand that was growing before the pandemic, addressing a growing health care delivery gap.^33^ It is even possible that the originating site model of centralized telemedicine facilities may be helpful to rural patients with audio-only mental health visits, which is another active area of telemedicine policy debate.^34,35^

We also found that telemedicine in rural communities was increasingly delivered by nonphysicians. This reflects the importance of NPs and other clinicians in rural areas as the shortage of physicians worsens in these communities.^36^ These NPs and PAs are also playing a growing role in key applications for telehealth, such as substance use disorder treatment.^37^ The prominence of nonphysicians as telemedicine clinicians should inform current policy development. For example, an ongoing policy debate regards the use of “incident to” billing for NPs and PAs, whereby these clinicians bill for services as if they were delivered by the supervising physician.^38^ This practice obscures the activity of nonphysician clinicians, and continued incident to billing will make it more challenging to track telemedicine trends and examples of fraud and abuse over time.

### Limitations

This analysis had several limitations. First, the study scope was limited to telemedicine delivery among the fee-for-service Medicare population. Therefore, conclusions from these results may not generalize to populations with other insurance coverage in the US, including Medicare Advantage and commercial insurance. However, as a large and vulnerable population of millions of older and disabled people in the US, telemedicine policy within fee-for-service Medicare has broad relevance even if it does not generalize elsewhere, and many insurance plans mirror Medicare’s standards for telemedicine coverage. Second, this study is descriptive and none of its conclusions should be interpreted as statements of causality. Another important issue to acknowledge is that by design, the study period does not cover the well-described changes in telemedicine use after the onset of the COVID-19 pandemic in the US. However, as telemedicine volumes decline and patients and clinicians become increasingly comfortable with in-person care and the permanency of payment changes remains debated, these trends show important considerations for telemedicine’s future within Medicare. An additional technical issue is that the study estimates of telemedicine delivered by NPs and PAs may be underestimated because of the commonly used “incident to” billing, which may obscure delivery of patient care in claims data.^39^ Finally, we are unable to determine the clinical appropriateness of care of telemedicine visits or patient satisfaction.

## Conclusions

This cross-sectional study found that before 2020 and the COVID-19 pandemic, there was sustained growth in telemedicine use among rural beneficiaries of the Medicare program, especially for visits with NPs and other nonphysician clinicians. Use of telemedicine was also concentrated within disadvantaged groups, such as patients with serious mental illness. In nearly 1 in 3 counties in the US, more than 10% of beneficiaries with serious mental illness used telemedicine in 2019. These findings imply that the telemedicine model used before the pandemic, in which patients receive treatment via telemedicine delivered in a local health care settings may be a viable model to maintain alongside the home-based telemedicine services that expanded during the COVID-19 pandemic.

## Notes

Supplement. **eTable 1. **Characteristics of all rural Medicare beneficiaries with and without a telemedicine visit in 2010, 2015, and 2019 **eFigure.** Trends in telemedicine visits for rural Medicare beneficiaries by patient characteristics, 2010 to 2019 **eTable 2. **Proportion of mental health and nonmental health telemedicine visits by health care professional’s specialty, 2010 to 2019
