# Supplementary material for: Trends in Outpatient Telemedicine Utilization Among Rural Medicare Beneficiaries, 2010 to 2019
Source: JAMA Health Forum. 2021 Oct 15;2(10):e213282. doi: 10.1001/jamahealthforum.2021.3282 (PMC8727042; doi:10.1001/jamahealthforum.2021.3282)
Supplement: Supplement. — eTable 1. Characteristics of all rural Medicare beneficiaries with and without a telemedicine visit in 2010, 2015, and 2019 eFigure. Trends in telemedicine visits for rural Medicare beneficiaries by patient characteristics, 2010 to 2019 eTable 2. Proportion of mental health and nonmental health telemedicine visits by health care professional’s specialty, 2010 to 2019 [file jamahealthforum-e213282-s001.pdf]

Supplemental Online Content

Barnett ML, Huskamp HA, Busch AB, Uscher-Pines L, Chaiyachati KH, Mehrotra A. Trends in outpatient telemedicine utilization among rural Medicare beneficiaries, 2010 to 2019. *JAMA Health Forum*. 2021;2(10):e213282. doi:10.1001/jamahealthforum.2021.3282

eTable 1. Characteristics of all rural Medicare beneficiaries with and without a telemedicine visit in 2010, 2015, and 2019
eFigure. Trends in telemedicine visits for rural Medicare beneficiaries by patient characteristics, 2010 to 2019
eTable 2. Proportion of mental health and nonmental health telemedicine visits by health care professional's specialty, 2010 to 2019

This supplemental material has been provided by the authors to give readers additional information about their work.

© 2021 Barnett ML, et al. *JAMA Health Forum.*

eTable 1. Characteristics of all rural Medicare beneficiaries with and without a telemedicine visit in 2010, 2015, and 2019

	TM Users			Non-TM Users		
	2010	2015	2019	2010	2015	2019
**Total**	17,214	47,463	91,483	10,161,464	10,454,543	10,329,920
**Age**						
<65	54.4	59.0	48.5	19.6	18.7	15.5
65-74	20.3	21.6	28.5	42.3	45.9	49.1
75-84	16.1	12.6	16.0	26.0	24.0	24.8
85+	9.3	6.7	7.0	12.1	11.4	10.6
**Race**						
Black	7.3	7.6	7.1	6.6	6.4	5.8
White	82.8	82.7	83.6	88.0	86.8	86.6
Other	9.9	9.6	9.4	5.4	6.7	7.6
**Sex**						
Male	41.6	43.7	43.7	46.6	47.6	48.2
Female	58.4	56.3	56.3	53.4	52.4	51.8
**Census Division**						
New England	1.2	1.5	2.8	4.0	4.2	4.1
Middle Atlantic	1.2	2.8	2.6	6.7	6.6	6.4
East North Central	11.2	14.2	17.4	16.1	15.3	15.4
West North Central	20.4	26.8	23.6	13.1	12.7	13.1
South Atlantic	13.7	13.5	14.0	19.0	18.9	18.5
East South Central	14.9	9.3	10.1	13.1	12.9	12.0
West South Central	22.0	15.8	11.9	13.8	13.4	12.9
Mountain	10.5	8.8	8.9	6.6	7.4	8.2
Pacific	4.9	7.5	8.8	7.6	8.6	9.4
**Medicare Eligibility**						
Age ≥65	36.9	30.3	38.0	71.9	71.8	74.7
Disability	62.5	69.0	61.4	27.4	27.6	24.8
End Stage Renal Disease	0.6	0.6	0.7	0.7	0.6	0.6
**Median ZIP Code Income**						
Less than 200% FPL	78.9	77.8	76.0	73.8	72.5	70.9
Greater than 200% FPL	21.1	22.2	24.0	26.2	27.5	29.1
**Medicaid Eligibility**						
Yes	60.6	63.9	56.9	22.8	21.3	18.6
No	39.4	36.1	43.1	77.2	78.7	81.4

Data on race came from an imputed variable created by the Research Triangle Institute for Medicare. “Other” race includes Asian, American Indian, Pacific Islander and Hispanic patients.

© 2021 Barnett ML, et al. *JAMA Health Forum.*

eFigure 1. Trends in telemedicine visits for rural Medicare beneficiaries by patient characteristics, 2010 to 2019

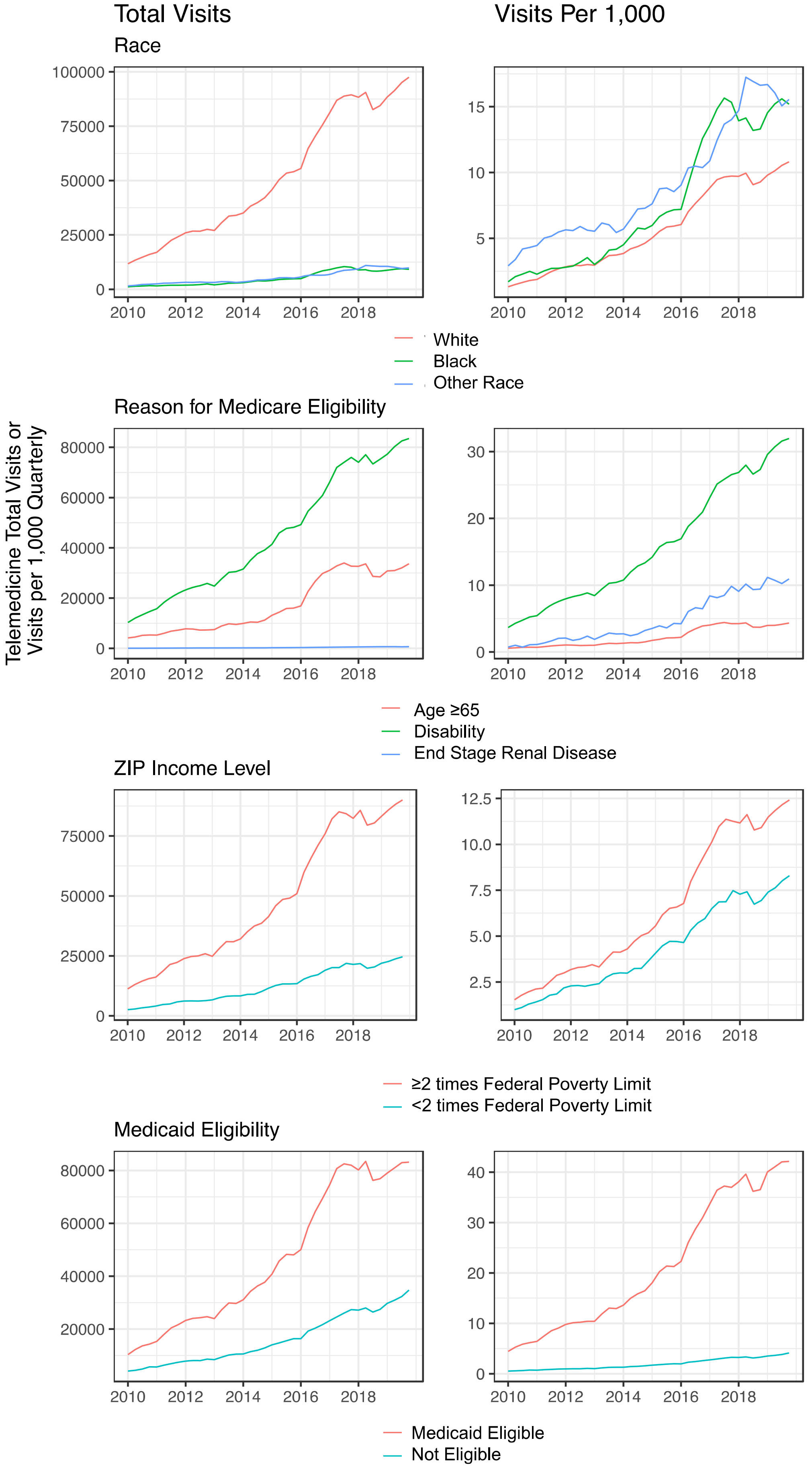

© 2021 Barnett ML, et al. *JAMA Health Forum.*

eTable 2. Proportion of mental health and nonmental health telemedicine visits by health care professional's specialty, 2010 to 2019

		Proportion of Visits (%)					
		Mental Health Telemedicine			Non-Mental Health Telemedicine		
		2010 (n=27,179)	2015 (n=108,291)	2019 (n=195,705)	2010 (n=15,429)	2015 (n=27,117)	2019 (n=62,274)
	Psychiatrist	71.2	59.2	41.0	18.5	17.5	5.4
	PCP	3.4	1.2	1.5	19.7	16.2	17.3
Non-MD Disciplines	LICSW	0.8	3.8	8.6	0.0	0.7	1.3
	Mental Health NP	7.0	20.7	27.0	1.7	4.5	3.6
	Nurse Practitioner	4.3	0.6	3.7	17.0	6.9	15.8
	PA	6.0	0.8	2.9	1.8	3.6	4.1
	Psychologist	3.4	13.3	14.9	0.2	4.5	5.5
Other Specialties	Cardiology	0.0	0.0	0.0	3.7	4.7	4.0
	Endocrine	0.0	0.0	0.0	2.2	3.5	3.9
	Heme-Onc	0.0	0.0	0.0	3.2	5.1	4.9
	ID	0.0	0.0	0.0	3.1	6.2	3.6
	Nephrology	0.0	0.0	0.0	2.3	4.0	5.2
	Neurology	0.0	0.0	0.0	4.4	4.3	4.5
	Pulmonary	0.0	0.0	0.0	3.0	2.4	3.1
	Sleep Medicine	0.0	0.0	0.0	0.0	3.6	5.5
	Other Specialists	4.0	0.4	0.4	19.1	12.4	12.2

Abbreviations: infectious disease (ID), licensed independent clinical social worker (LICSW), nurse practitioner (NP), physician assistant (PA), primary care physician (PCP)

© 2021 Barnett ML, et al. *JAMA Health Forum.*
